# Modification of Pulsed Electric Field Conditions Results in Distinct Activation Profiles of Platelet-Rich Plasma

**DOI:** 10.1371/journal.pone.0160933

**Published:** 2016-08-24

**Authors:** Andrew L. Frelinger, Anja J. Gerrits, Allen L. Garner, Andrew S. Torres, Antonio Caiafa, Christine A. Morton, Michelle A. Berny-Lang, Sabrina L. Carmichael, V. Bogdan Neculaes, Alan D. Michelson

**Affiliations:** 1 Center for Platelet Research Studies, Division of Hematology/Oncology, Boston Children's Hospital, Dana-Farber Cancer Institute, Harvard Medical School, Boston, Massachusetts, United States of America; 2 School of Nuclear Engineering, Purdue University, West Lafayette, Indiana, United States of America; 3 GE Global Research Center, Niskayuna, New York, United States of America; Emory University/Georgia Institute of Technology, UNITED STATES

## Abstract

**Background:**

Activated autologous platelet-rich plasma (PRP) used in therapeutic wound healing applications is poorly characterized and standardized. Using pulsed electric fields (PEF) to activate platelets may reduce variability and eliminate complications associated with the use of bovine thrombin. We previously reported that exposing PRP to sub-microsecond duration, high electric field (SMHEF) pulses generates a greater number of platelet-derived microparticles, increased expression of prothrombotic platelet surfaces, and differential release of growth factors compared to thrombin. Moreover, the platelet releasate produced by SMHEF pulses induced greater cell proliferation than plasma.

**Aims:**

To determine whether sub-microsecond duration, low electric field (SMLEF) bipolar pulses results in differential activation of PRP compared to SMHEF, with respect to profiles of activation markers, growth factor release, and cell proliferation capacity.

**Methods:**

PRP activation by SMLEF bipolar pulses was compared to SMHEF pulses and bovine thrombin. PRP was prepared using the Harvest SmartPreP2 System from acid citrate dextrose anticoagulated healthy donor blood. PEF activation by either SMHEF or SMLEF pulses was performed using a standard electroporation cuvette preloaded with CaCl_2_ and a prototype instrument designed to take into account the electrical properties of PRP. Flow cytometry was used to assess platelet surface P-selectin expression, and annexin V binding. Platelet-derived growth factor (PDGF), vascular endothelial growth factor (VEGF), endothelial growth factor (EGF) and platelet factor 4 (PF4), and were measured by ELISA. The ability of supernatants to stimulate proliferation of human epithelial cells in culture was also evaluated. Controls included vehicle-treated, unactivated PRP and PRP with 10 mM CaCl_2_ activated with 1 U/mL bovine thrombin.

**Results:**

PRP activated with SMLEF bipolar pulses or thrombin had similar light scatter profiles, consistent with the presence of platelet-derived microparticles, platelets, and platelet aggregates whereas SMHEF pulses primarily resulted in platelet-derived microparticles. Microparticles and platelets in PRP activated with SMLEF bipolar pulses had significantly lower annexin V-positivity than those following SMHEF activation. In contrast, the % P-selectin positivity and surface P-selectin expression (MFI) for platelets and microparticles in SMLEF bipolar pulse activated PRP was significantly higher than that in SMHEF-activated PRP, but not significantly different from that produced by thrombin activation. Higher levels of EGF were observed following either SMLEF bipolar pulses or SMHEF pulses of PRP than after bovine thrombin activation while VEGF, PDGF, and PF4 levels were similar with all three activating conditions. Cell proliferation was significantly increased by releasates of both SMLEF bipolar pulse and SMHEF pulse activated PRP compared to plasma alone.

**Conclusions:**

PEF activation of PRP at bipolar low *vs*. monopolar high field strength results in differential platelet-derived microparticle production and activation of platelet surface procoagulant markers while inducing similar release of growth factors and similar capacity to induce cell proliferation. Stimulation of PRP with SMLEF bipolar pulses is gentler than SMHEF pulses, resulting in less platelet microparticle generation but with overall activation levels similar to that obtained with thrombin. These results suggest that PEF provides the means to alter, in a controlled fashion, PRP properties thereby enabling evaluation of their effects on wound healing and clinical outcomes.

## Introduction

Platelet gel is a substance derived from platelet-rich plasma (PRP), which contains a concentrated amount of platelets that can be activated to release proteins and growth factors found within the alpha granules. These growth factors have various beneficial effects, such as angiogenesis and tissue regeneration [1,2]. Autologous platelet gel can enhance wound healing [2,3], induce hemostasis [4], and provide antibacterial protection for the wound as it heals [5].

The typical workflow for generating autologous platelet gel includes an intravenous blood draw from the patient, platelet enrichment using commercially available kits, and then platelet activation. Currently, platelet activation is performed using the protein bovine thrombin (in the USA) or other types of thrombin in Canada and Europe (recombinant thrombin or thrombin from human donor plasma) [3,6,7]. These various types of thrombin in current use are expensive, may trigger significant side effects and must be stored under refrigeration. Moreover, bovine thrombin can stimulate antibody formation, potentially inducing severe hemorrhagic or thrombotic complications or an allergic response in patients previously exposed to bovine thrombin [8–10]. Approximately 30% of patients exposed to bovine thrombin develop cross-reacting antibodies [11]. Thus, some clinicians consider that using bovine thrombin as the activator for repeated applications of PRP for wound healing introduces unacceptably high risk [12]. This motivates the exploration of a physical means of platelet activation not requiring an external agent.

Electric pulse stimulation using nanosecond pulsed electric fields (PEF) is an alternative, non-biochemical method of platelet activation [13–15] that avoids exposure to xenogeneic thrombin with its associated risks. PEFs can induce multiple changes in biological function depending upon pulse duration and intensity [16–18]. Applying PEFs with durations on the order of microseconds to milliseconds with electric fields of hundreds of V/cm to a few kV/cm permeabilizes the plasma membrane in a process called electroporation [16,19]. The resulting pores may grow so large that they cannot reseal upon removal of the PEF, inducing cell death by irreversible electroporation, which is used in cancer treatment [20,21] and liquid sterilization [22]. Alternatively, appropriate selection of the pulse duration and intensity may permit the pores to reseal after the pulse ends, enabling molecular delivery while retaining viability. This reversible electroporation may be used for electrochemotherapy [23] or as a physical method of gene therapy [24]. Recent studies have explored the impact of applying PEFs with the same total energy over a shorter duration (10–300 ns) with higher intensities (30–300 kV/cm) [16,25,26]. These nanosecond PEFs (nsPEFs) fully charge the membranes of intracellular organelles prior to the plasma membrane, paving the way for intracellular effects, such as releasing intracellular calcium stores [27], permeabilizing intracellular structures [28], and inducing apoptosis [29], without permeabilizing the plasma membrane to standard dyes for membrane integrity, such as propidium iodide and ethidium homodimer. Subsequent studies using the smaller dye YO-PRO1 [30,31] and electrical measurements [32] demonstrated that nsPEFs still permeabilize the plasma membrane, but with pores much smaller than conventional electroporation. Interestingly, applying multiple nsPEFs creates the number of long-lived plasma membrane pores, but not the size [33]. Additionally, the intense electric field concomitant with nsPEF application contributes an electrophoretic effect to ion delivery, as demonstrated experimentally [34], by molecular dynamics simulations [34], and through modeling studies [35]. The capability to transport ions without creating large membrane pores that could induce cell death by irreversible electroporation facilitates applications requiring ion transport with minimal long-term plasma membrane damage, such as nervous system manipulation [36].

Calcium transport plays a critical role in platelet activation, motivating initial studies in using nsPEFs for platelet activation [13]. We previously demonstrated PEF-stimulated release of growth factors from PRP prepared from outdated platelets, aged blood [13,15] and fresh blood [37] using an automated centrifugation system standardized for clinical use. Compared to thrombin, exposure of PRP to sub-microsecond duration, high electric field (SMHEF) pulses induced greater generation of microparticles and expression of prothrombotic platelet surfaces, and differential release of growth factors [37]. Moreover, the platelet releasate produced by SMHEF pulses induced greater cell proliferation than plasma. Our previous work on platelet activation has used monopolar SMHEF pulses only [14,15,37]. However, exposure of other cell types to SMHEF often results in cell death by apoptosis [38,39]. Additionally, the SMHEFs may induce other effects, such as externalization of phosphatidylserine, a lipid that is normally on the inside of the lipid bilayer and is externalized during apoptosis, by physically altering the plasma membrane [40].

Many questions remain regarding the mechanism of PRP activation by SMHEF and whether other electric pulse parameters would yield the same result. SMHEF can rapidly and transiently release Ca^2+^ from intracellular stores [41,42], which is hypothesized to occur due to ER permeabilization and subsequent diffusion of Ca^2+^ down its electrochemical gradient into the cytosol. This intracellular release of Ca^2+^ has most closely been associated with Ca^2+^-mediated intracellular signaling and has been demonstrated to activate platelets [13]. In general, higher power nanosecond duration pulses are thought to preferentially breach smaller structures, such as intracellular organelles, while minimally impacting the plasma membrane [43], although studies have demonstrated that these pulses can induce nanopores that are smaller than those induced by conventional electroporation [30,31,44].

Alternatively, one may apply bipolar pulses, which enhance membrane permeabilization and delivery efficiency for microsecond duration pulses. Bipolar pulses consist of a pulse of one polarity (positive or negative) followed by a pulse of the reverse polarity either immediately or following some time lag after the first pulse, but induce different effects depending upon the pulse duration and time between pulses. Applying bipolar pulses in the microsecond regime induced improved transfection efficiency with reduced cell death [45,46]; however, bipolar nanosecond pulses actually induce effect reversal [44,47,48]. In other words, bipolar nanosecond pulses of high intensity induce less membrane permeabilization, ion transport, or cell death than nanosecond pulses with either the same duration as a single pulse or a the same duration as the combined overall duration of the two bipolar pulses. Introducing increasing delays between the bipolar pulses reduced the cancellation effects, but they were still visible for delays up to 10 μs [48]. The reason for the difference in behavior between bipolar nanosecond and microsecond pulses is currently unclear. One potential explanation is that nanosecond pulses induce nanopores and the major impact of ion transport on nanosecond timescales is electrophoresis [35]. Applying another nanosecond pulse a very short time (say, within hundreds of nanoseconds) may induce a reversal of this electrophoretic ion motion and cancel the biological effects if diffusion and electrophoresis are at least equally important on long timescales, which calculations suggest may be the case [44][44]. While this may explain the reversal in ion motion, it does not necessarily completely explain the change in cell death induction by bipolar nanosecond duration pulses. Recent experiments and finite element simulations have explored the impact of nanosecond pulse induced shock waves on biological cells [49–51]. Shock waves would create mechanical stresses on the biological cells that could be reversed by the application of opposite polarity pulses within a short period of time [51]. The long-term effects of bipolar pulses also remain incompletely understood. Electrophoresis clearly dominates ion motion during the pulse; long-term ion motion (on the order of hundreds of microseconds and longer) is actually diffusion through long-lived pores with lifetimes ranging from hundreds of nanoseconds to tens of minutes. The impact of these contributions can be clearly seen through simulations [35]. Thus, even if bipolar pulses reverse electrophoretic motion *during* the pulse, one would anticipate that long-term ion diffusion into the cell could still occur. While calculations indicate that electrophoresis and diffusion are approximately equally important [44], this will likely vary quite significantly with pulse parameters, including duration, intensity, and delay between pulses, which can impact pore size and lifetime in addition to diffusion and electrophoresis. Future studies may further elucidate the impact of bipolar pulse parameters on the ion transport, which could be particularly important for platelet activation.

The nanosecond electric pulses studies discussed above explore the impact of *high intensity* submicrosecond electric pulses. Alternatively, one may consider the membrane level effects of *low intensity* submicrosecond (SMLEF) bipolar pulses. In this case, one would not anticipate the potential induction of shock waves observed for the higher intensity bipolar submicrosecond pulses or the same level of plasma membrane permeabilization. One may, however, still induce some degree of intracellular manipulation of the ER while controlling ion flow and minimizing adverse effects on viability and morphology. The present study evaluates platelet activation and procoagulant markers, growth factor release, and the capacity of the treated PRP to induce cell proliferation following the application of SMLEF bipolar pulses, SMHEP monopolar pulses, and bovine thrombin to fresh PRP prepared using a clinically relevant centrifugation procedure.

## Materials and Methods

### Donors, Blood Collection and Preparation of PRP

This study was reviewed and approved by the Boston Children’s Hospital Committee on Clinical Investigation and all subjects provided written informed consent. Healthy volunteers were qualified for enrollment if they were aged ≥18 years, free of aspirin or other antiplatelet medication (≥10 days), and free of all other non-steroidal anti-inflammatory drugs (≥ 3 days). Blood, 120 mL, was collected into 1/10^th^ volume of acid citrate dextrose (ACD) and PRP was prepared Harvest SmartPreP2 System (Harvest Technologies, Plymouth, MA, USA) according to the manufacturer’s recommendation as previously described [37]. Complete blood cell counts were performed in a Sysmex XE-2100 Hematology Analyzer. Prepared PRP had 1095.2 ± 192.9 x 10^9^ platelets/L, 1.65 ± 0.26 ×10^12^ RBC/L, and 13.77 ± 3.98 x 10^9^ WBC/L (mean ± SD).

### Study Design

The ability of SMLEF bipolar pulses to activate concentrated PRP was compared to SMHEF pulses and bovine thrombin (1 U/mL final concentration, Biopharm Laboratories LLC, Bluffdale, UT, USA) PRP activation as measured by platelet surface P-selectin expression, platelet-derived microparticle generation, platelet and microparticle surface phosphatidylserine expression, growth factor release, and the capacity of the treated PRP to induce cell proliferation. PRP samples were recalcified by addition of 1/100^th^ volume of CaCl_2_ (10 mM final concentration, Bachem, Torrance, CA, USA) immediately prior to activation with SMHEF pulse, SMLEF bipolar pulse or thrombin. Control samples were treated with vehicle (0.9% NaCl) without prior recalcification and without electrical activation. To allow recovery of platelets for assessment of platelet activation markers by flow cytometry, clotting was prevented in electrically stimulated and thrombin-treated PRP samples by mixing Gly-Pro-Arg-Pro (GPRP, 2 μL, 40 mM final concentration) with a small portion of PRP (18 μL) immediately after activation. The remainder of the sample was allowed to stand 15 min at RT following activation, then clots were removed using the wooden handle of a cotton swab and the resulting serum was frozen at -80°C for later evaluation of released growth factors and cell proliferation activity.

### SMLEF Bipolar Pulse and SMHEF Pulse Stimulation of PRP

**Fig 1:**
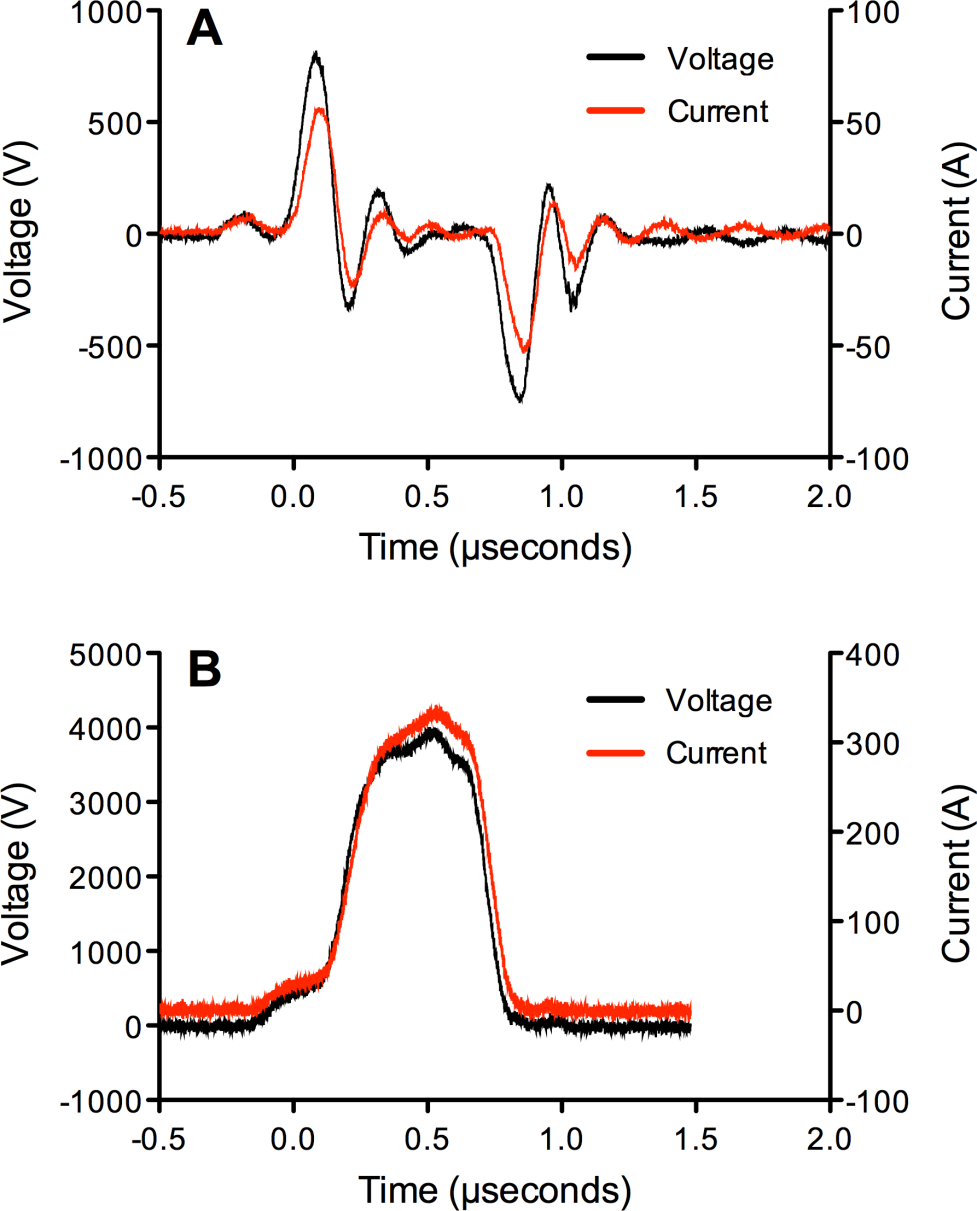
**Representative electrical tracings for A) SMLEF bipolar pulse and B) SMHEF monopolar pulse.** A) SMLEF bipolar pulse was ~150 ns pulse width, ~650 ns interval between pulses of opposite polarity,~4kV/cm electric field. B) SMHEF monopolar pulse was pulse ~650 ns, 20 kV/cm electric field. Samples received a total of 80 pairs of bipolar SMLEF pulses at 1 second intervals (the spacing between the opposite polarity pulses within a pair of bipolar pulses was about 650 ns, as shown in Fig 1A; a pair of two bipolar pulses, as shown in Fig 1A, was applied every 1 second–a total of 80 pairs) or 5 monopolar pulses at 1 second intervals. Black tracing: voltage; red tracing: current.

Electrical stimulation of PRP was performed using a specially designed instrument prototype (GE Global Research, Niskayuna, NY, USA), which has previously been described [15,52]. For generation of bipolar pulses, a capacitor was placed between the instrument output and the cuvette. The instrument takes into account the specific electrical properties of PRP which is typically more conductive than the buffers used in electroporation. Concentrated PRP (400 μL) was placed in a 2 mm electroporation cuvette (Molecular BioProducts, San Diego, CA, USA), containing 1/100^th^ volume CaCl_2_ (10 mM final concentration), then exposed to SMLEF bipolar pulses (bipolar pulses, 150 ns pulse width, time delay between two bipolar pulses was about 500 ns, 80 pairs of bipolar pulses, ~4kV/cm electric field) or SMHEF monopolar pulses (five electric field pulses, one pulse per second; pulse widths ~500 ns, 20 kV/cm electric field e.g., ~ 5-fold higher than SMHEF, resulting in ~300 A current). A Tektronix DPO4104 oscilloscope and a Tektronix P6015A high voltage probe were used to measure the voltage pulses applied to cuvettes with PRP for activation. **Fig 1** shows an example of SMLEF bipolar pulse and SMHEF pulse used for the platelet activation experiments described herein.

### Characterization of Platelet Activation and Procoagulant Markers by Flow Cytometry

Platelets and platelet-derived microparticles were identified and enumerated by flow cytometry on the basis of surface CD41 expression and particle forward light scatter (a reflection of particle size), side light scatter (a reflection of granularity) as previously described [37,53,54]. Briefly, activated and control PRP samples were diluted 10-fold in HEPES-Tyrode’s buffer with 0.35% bovine serum albumin (HT-BSA; 10 mM HEPES, 7 mM NaCl, 2.8 mM KCl, 1 mM MgCl_2_, 12 mM NaHCO_3_, 0.4 mM Na_2_HPO_4_, 5.5 mM glucose, 0.35% bovine serum albumin; chemicals from Sigma, St. Louis, MO, USA) then added to a mixture of phycoerythrin (PE)-conjugated anti-CD62P (clone AK4, BD Pharmingen, San Diego, CA, USA) and CD41-PerCP-Cy5.5 (clone HIP8, BD Pharmingen, San Diego, CA, USA). After 15 min at room temperature, the reaction was stopped by fixation with 1 mL 1% formaldehyde, 10 mM HEPES, 0.15 M NaCl, pH 7.4. Platelet and microparticle counts were determined in samples mixed with calibrated counting beads (Spherotech Inc., Libertyville, IL). Flow cytometric analysis was performed in a calibrated standard configuration Becton Dickinson FACSCalibur equipped with a 488 nm laser. Control samples of platelets labeled with each individual fluorescent antibody were used to set hardware compensation and account for spectral overlap. In particular, compensation was adjusted so that the fluorescence in the PE channel (FL2) of platelets stained with CD41-PerCP-Cy5.5 (FL3) was identical to the fluorescence observed for platelets stained only with PE-conjugated normal IgG. Final color compensation settings were as follows: FL1–1.4% FL2, FL2–9.3% FL1, FL2–6.9% FL3, FL3–14.2% FL2. The threshold was set on FL3 to include only those events labeling positively for CD41. Platelets were identified by means of CD41-PerCP-Cy5.5 positivity and characteristic logarithmic forward and orthogonal light scatter. CD41-positive events with lower forward light scatter than characteristic of platelets were defined as platelet-derived microparticles and CD41-positive events with higher forward light scatter than platelets were defined as aggregated platelets. Non-specific staining was determined in parallel using a sample reacted with a mixture of isotype-matched PE and PerCP-Cy5.5-conjugated normal immunoglobulin.

Phosphatidylserine expression on platelets and platelet-derived microparticles were determined by annexin V binding and light scatter, as previously described [37,55,56]. Briefly, treated PRP samples were diluted 20-fold in HT-BSA with GPRP (50 μM final), incubated 15 min at room temperature with FITC-conjugated annexin V, PE anti-CD41 (clone HIP8, BD Pharmingen, San Diego, CA, USA) and a PE-Cy5 anti-CD42b antibody (as a platelet identifier; both reagents from BD Biosciences, San Jose, CA, USA) in the presence or absence of CaCl_2_ 4 mM, then fixed by addition of 1 mL 1% formaldehyde in HEPES-saline. Flow cytometric analysis was performed in a calibrated Becton Dickinson FACSCalibur with the threshold set on CD41 positive events to identify platelet-related events and then gating on platelet and platelet-derived microparticle populations according to their light scatter properties. Hardware compensation was set based on samples stained with individual fluorophores.

### Growth Factor Release

Commercially available ELISA kits were used to measure platelet-derived growth factor (PDGF, R&D Systems, Minneapolis, MN, USA), vascular endothelial growth factor (VEGF, Eagle Biosciences, Nashua, NH, USA), endothelial growth factor (EGF, R&D Systems, Minneapolis, MN, USA) and platelet factor 4 (PF4, Abcam, Cambridge, UK) in supernatants of activated and control PRP.

### Cell Proliferation

Supernatants of activated PRP was evaluated for their ability to stimulate proliferation of a human non-tumorigenic epithelial line (MCF 10A [ATCC® CRL-10317™], American Type Culture Collection, Manassas, VA, USA) [[57,58] as previously described [37]. Briefly, MCF 10A cells seeded at 200,000 cells/cm^2^ in McCoy’s medium supplemented with 10% fetal bovine serum (Invitrogen, Grand Island, NY, USA), were grown for 24 hours at 37°C in 5% CO_2_ then washed twice with Hank’s Balanced Salt Solution (Invitrogen) and placed in serum-free media for an additional 24 hours. The serum-starved cells were then incubated for 24 hours at 37°C with control PPP (100 μL) from unactivated PRP or the supernatants of PEF- or thrombin-treated PRP. Cell proliferation was monitored by measuring total ATP/well using the ATPlite 1step single addition luminescence ATP detection assay (Perkin Elmer, Waltham, MA, USA) according to the manufacturer’s recommendations. Growth factor-dependent cell proliferation was confirmed by addition of purified recombinant human EGF (Lonza, Portsmouth, NH, USA).

### Statistical Analysis

Data were analyzed using SAS software, version 9.2 (SAS Institute, Cary, NC, USA) and GraphPad Prism version 5.0a (GraphPad Software, La Jolla, CA, USA). Normally distributed data (as judged by the D’Agostino and Pearson omnibus normality test) are summarized as mean ± standard deviation or mean ± standard error of the mean, as indicated. Non-parametric data are reported as median and interquartile range or median and range.

## Results

### Flow Cytometric Analysis of Platelets and Platelet-Derived Microparticles

**Fig 2:**
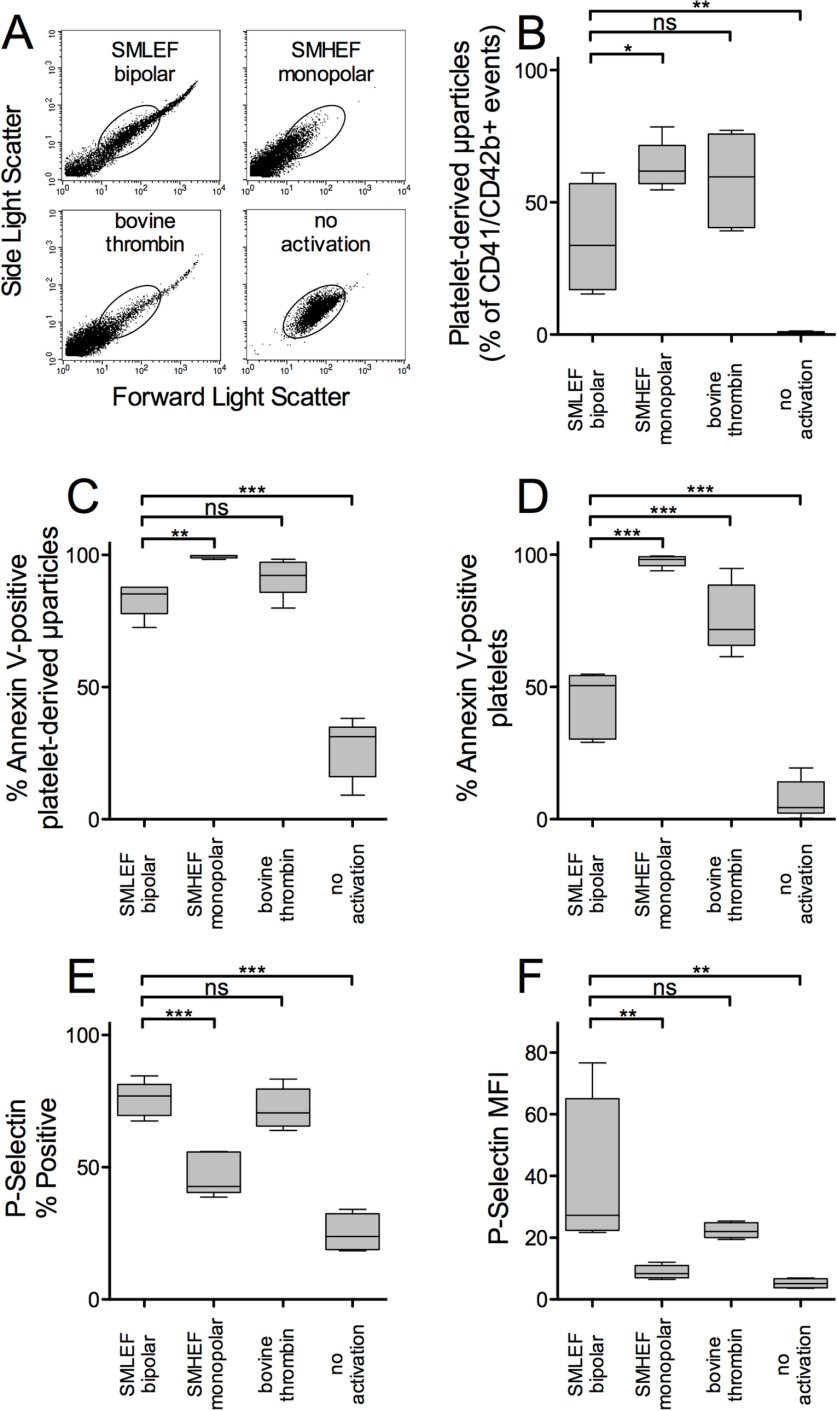
Flow cytometric analysis of platelets and platelet-derived microparticles (PDMP) in PRP following activation with SMLEF bipolar pulses, SMHEF monopolar pulses and thrombin. A) Representative forward- and side-light scatter profiles of (CD41/CD42b double positive) particles in activated and unactivated PRP samples. The oval indicates the location of the normal forward and side-light scatter distribution for intact platelets; CD41+/CD42b+ particles with lower forward and side light scatter are considered PDMP. B) PDMP as % of all CD41/CD42b double positive particles. Platelet count prior to stimulation was 1095.2 ± 192.9 x 10^9^/L (mean ± SD). C) Percentage of PDMP positive for surface phosphatidylserine as detected by annexin V binding; D) Percentage of platelets positive for surface phosphatidylserine as detected by annexin V binding; E) Percentage of all CD41/CD42b double positive particles positive for surface P-selectin. F) P-selectin mean fluorescence intensity (MFI) per particle. Upper and lower boundaries of boxes represent 25^th^ and 75^th^ %tile, whiskers represent 10^th^ and 90^th^ %tiles, line indicates median, n = 5. *p<0.05, **p<0.01, ***p<0.001.

Flow cytometric analysis of PRP treated with SMLEF bipolar pulse or with bovine thrombin showed forward-light scatter (FSC) and side-light scatter (SSC) profiles consistent with the presence of platelet-derived microparticles (low FSC and SSC), platelet-sized particles (medium FSC and SSC), and platelet-platelet aggregates (high FSC and SSC) (Fig 2A). In contrast, SMHEF pulses produced few platelet-platelet aggregates (high FSC and SSC) and significantly more platelet-derived microparticles (as a percent of all CD41/CD42b positive events) than SMLEF bipolar pulses (Fig 2A and 2B). In addition to SMLEF bipolar pulses producing fewer microparticles than SMHEF pulses, those that were produced were less procoagulant as judged by % annexin V-positivity (Fig 2C). The % of procoagulant annexin V-positive platelets (medium FSC and SSC events) was lower with SMLEF bipolar pulses than with either SMHEF or bovine thrombin (Fig 2D).

While annexin V-positivity for microparticles and platelets in SMLEF bipolar pulses was lower than that for SMHEF, the % P-selectin positivity and surface P-selectin expression (MFI) for platelets and microparticles in SMLEF bipolar pulse activated PRP was significantly higher than that in SMHEF-activated PRP, but not significantly different from that produced by thrombin activation (Fig 2E and 2F). These results are summarized in **Table 1**and detailed in S1–S10 Tables.

**Table 1:** Differential effects of SMLEF bipolar pulses, SMHEF pulses and thrombin on PRP activation, growth factor release, and cell proliferation.

	SMLEF bipolar	SMHEF monopolar	Thrombin	no activation	SMLEF bipolar *vs*. SMHEF monopolar	SMLEF bipolar *vs*. Thrombin	SMLEF bipolar *vs*. no activation
**PDMP (% of CD41/CD42b+ events)**	36.4 ± 9.1	63.8 ± 4.0	58.4 ± 7.9	0.82 ± 0.16	*	ns	**
**Annexin V-positive PDMP (%)**	83.3 ± 2.8	99.4 ± 0.28	91.70± 3.2	26.7 ± 5.0	**	ns	***
**Annexin V-positive platelets (%)**	44.0 ± 5.6	97.7 ± 0.99	76.0 ± 5.7	7.5 ± 3.3	***	***	***
**Platelet surface P-selectin (% positive platelets)**	75.8 ± 2.9	47.0 ± 3.6	72.2 ± 3.4	38.1 ± 13.3	***	ns	***
**Platelet surface P-selectin (MFI)**	38.2 ± 12.9	9.0 ± 1.2	21.9 ± 1.27	5.2 ± 0.78	**	ns	**
**EGF (ng/mL)**	2.36 ± 0.27	2.90 ± 0.28	1.43 ± 0.17	0.02 ± 0.005	ns	*	***
**VEGF (pg/mL)**	783 ± 200	773 ± 154	633 ± 195	62.5 ± 0.00	ns	ns	*
**PDGF (ng/mL)**	15.1 ± 3.0	11.1 ± 1.4	14.1 ± 2.9	0.32 ± 0.07	ns	ns	***
**PF4 (μg/mL)**	20.5 ± 3.7	14.8 ± 3.2	20.1 ± 2.7	0.34 ± 0.04	ns	ns	**
**Cell proliferation (normalized)**	1.20 ± 0.05	1.19 ± 0.05	1.14 ± 0.06	1.00 ± 0.04	ns	ns	*

The indicated parameters were measured in PRP exposed to SMLEF bipolar pulses, SMHEF monopolar pulses, bovine thrombin, or no activator. Results for cell proliferation in response to the plasma supernatants of activated PRP are shown normalized to the cell proliferation obtained with plasma from unactivated PRP. Purified recombinant human EGF 100 ng/mL added to serum-free media increased cell proliferation 1.75-fold relative to media alone (data not shown). Results shown are means ± SEM, n = 5

*p<0.05

**p<0.01

***p<0.001 by Dunnett’s multiple comparison test. Abbreviations: EGF, epidermal growth factor; MFI, mean fluorescence intensity; SMHEF, sub-microsecond high electric field; SMLEF, sub-microsecond low electric field; PDGF, platelet-derived growth factor; PF4, platelet factor 4; VEGF, vascular endothelial growth factor.

### Growth Factor Release and Cell Proliferation

**Fig 3:**
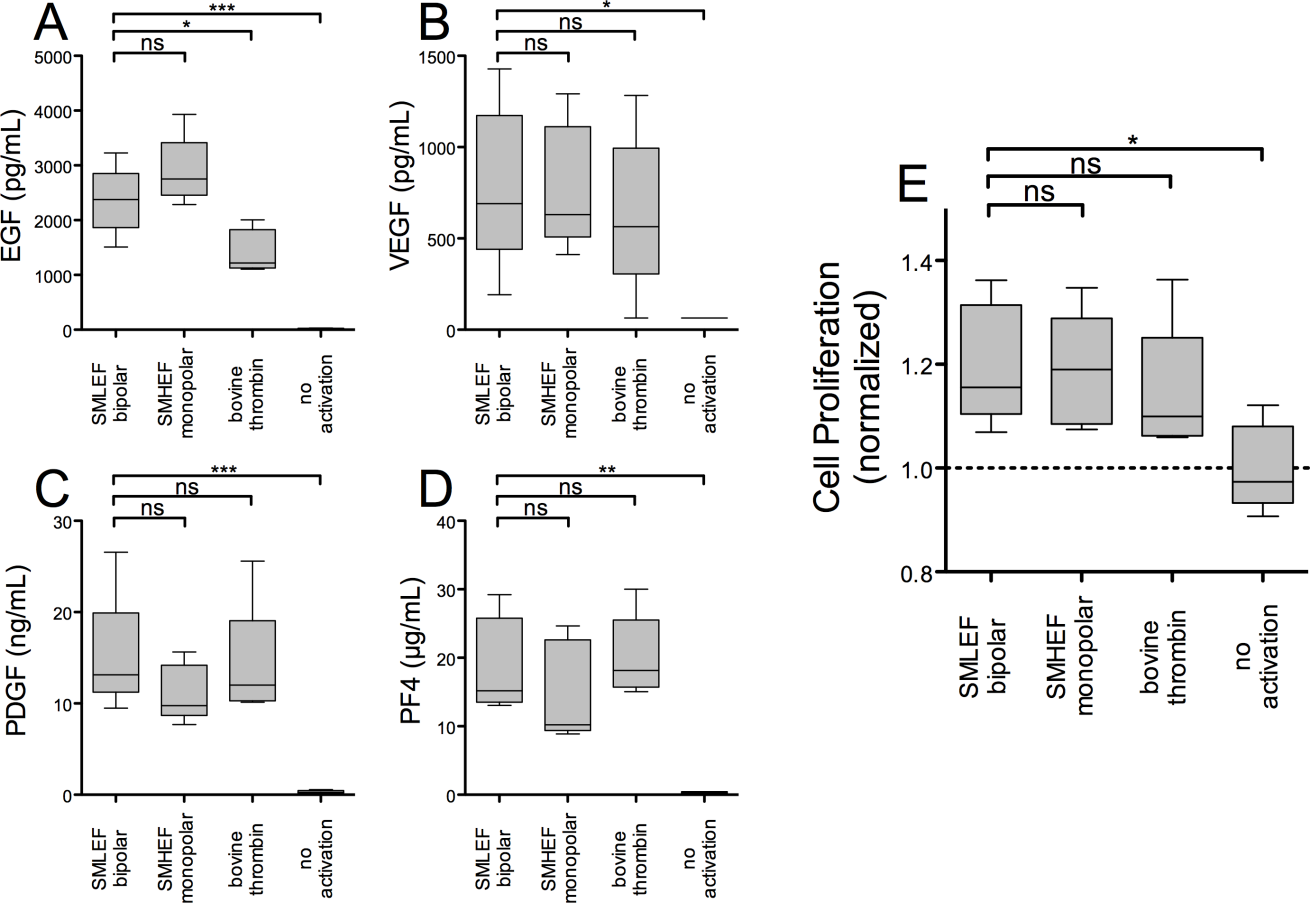
Growth factor release and stimulation of cell proliferation. PRP, treated as described in the Methods, was centrifuged, the supernatant recovered and assayed for pro- and anti-angiogenic factors by ELISA and for stimulation of cell proliferation using serum-starved epithelial cells (MCF10A). Cell proliferation in response to the plasma supernatants of unactivated or activated PRP (panel E) is normalized to that obtained with supernatants of unactivated PRP. Purified recombinant human EGF 100 ng/mL added to serum-free media increased cell proliferation 1.75-fold relative to media alone (data not shown). Results shown are means ± SEM, n = 5. *p<0.05, **p<0.01, ***p<0.001.

EGF, VEGF, PDGF, and PF4 in the supernatants of SMLEF bipolar pulse-treated PRP were significantly elevated compared to supernatants of unactivated PRP, but were not significantly different from levels in supernatants of SMHEF pulse-treated PRP (Fig 3A–3D). Compared to thrombin, SMLEF bipolar pulse resulted in similar levels of VEGF, PDGF and PF4 and significantly higher levels of EGF (Fig 3A–3D, Table 1).

Proliferation and survival of MCF10 cells, an epithelial cell line, as judged by luminescent measurement of total ATP, was increased 1.75-fold by addition of EGF 100 ng/mL compared to proliferation in serum-free media. Supernatants of PRP stimulated with SMLEF bipolar pulse and SMHEF pulse both significantly increased the proliferation of MCF10 cells in culture (~1.2-fold) compared to the proliferation seen with plasma alone (Fig 3E, Table 1). This proliferation was not significantly greater than that produced by supernatants of bovine thrombin-treated PRP (Fig 3E, Table 1). The higher relative cell proliferation seen with purified EGF (~75% increase in ATP) compared to that seen with activated PRP supernatants (~20% increase in ATP), may in part be explained by the much higher concentration of purified EGF added to cultures (100 ng/mL) compared to the amount of EGF found in the supernatants of activated PRP (up to 4 ng/mL, Fig 3, Table 1). Growth factor and cell proliferation results for individual donors are provided in S1–S10 Tables.

## Discussion

SMLEF bipolar pulse activation of PRP, compared to activation of PRP with SMHEF pulse conditions, preserves platelet size (as judged by forward light scatter) and yields fewer microparticles (Fig 2A and 2B). The resulting particles have lower phosphatidylserine expression and higher surface P-selectin expression, which may favor their participation in inflammatory processes more than procoagulant processes compared to particles generated by SMHEF pulse treatment of PRP. Nevertheless, growth factor release was similar for the two conditions, as was net cell proliferation. (The end point for the proliferation assay, total ATP, is the net result of cell proliferation and cell death or apoptosis.) Further study is required to determine whether the differences in the characteristics of PRP activated by these methods will have differential effects on specific phases of wound healing.

The mechanism of platelet activation following nanosecond PEF stimulation is poorly understood. In general, higher power nanosecond duration pulses are thought to preferentially breach smaller structures, such as intracellular organelles, with less impact on the plasma membrane [16,23]. Zhang *et al*.[13] reported that platelets exposed to monopolar high electric field nanosecond pulses showed increases in cytosolic calcium (Ca^2+^) that were dose-dependent on the electrical energy density of the pulses and hypothesized that nanopore formation in organelle membranes and the plasma membrane allowed Ca^2+^ leakage from intracellular stores and an influx of extracellular Ca^2+^. This intracellular release of Ca^2+^ has most closely been associated with Ca^2+^-mediated intracellular signaling and has been demonstrated to activate platelets [13]. However, such monopolar pulses also induce electrophoretic transport of proteins and ions, particularly Ca^2+^ [37,59]. In contrast, bipolar nanosecond pulses (with proper delay between pulses) reverse electrophoretic transfer [44,47,48], generally cancelling ion transport and changes in viability compared to monopolar nanosecond pulses [47,48]. However, as noted above, these nanosecond bipolar pulses may also induce shock waves [49–51] that induce mechanical, in addition to electrical, effects on the cells. These mechanical effects on the cell may subsequently be cancelled if the delay between first and second pulse is sufficiently short, as observed experimentally by the reduction in effect cancellation for increasing delay times [48]. While these previous studies considered the impact of *high* intensity bipolar pulses, the present study explores *low* intensity bipolar effects, or SMLEF bipolar pulses, with a short delay between the bipolar pulses (~ 500 ns). These lower intensity fields would not be anticipated to induce strong shock waves and may not be victim to the same cancellation observed in the previous studies. Thus, the present study compares PRP activation using SMLEF bipolar pulses with SMHEF monopolar pulses. Interestingly, SMLEF bipolar pulses induce similar levels of growth factor release as monopolar SMHEFs, which contradicts the effect reversal observed for SMHEF bipolar pulses, suggesting that the intensity of the fields plays an important role in effect cancellation. Future research could further elucidate the importance of pulse parameters on bipolar nanosecond pulse effects, particularly for low intensity fields, which have not been studied in detail.

As mentioned, SMLEF bipolar pulses and SMHEF pulses stimulated release of similar levels of the four growth factors evaluated: EGF, VEGF, PDGF, and PF4, but both SMLEF and SMHEF stimulated greater release of the proangiogenic growth factor EGF than bovine thrombin (Fig 3A). The release of similar levels of growth factors following SMLEF bipolar pulses and SMHEF pulses is somewhat surprising, given that the growth factors are primarily stored in platelet alpha granules [1] and P-selectin, a marker of platelet alpha granule release [60], was significantly higher on platelets and microparticles exposed to SMLEF bipolar pulses than on those following SMHEF monopolar pulses. One possible explanation for this is that both SMLEF bipolar pulses and SMHEF pulses stimulate virtually complete alpha granule release, and thus similar levels of growth factors, but that SMHEF monopolar pulses induce further changes which result in increased microparticle formation; with smaller particles carrying less P-selectin. Indeed, annexin V-positive microparticles were increased with SMHEF monopolar pulses compared to SMLEF bipolar pulses. While growth factors are recognized to be important in the proliferation phase of wound healing [61,62], EGF is particularly important for epithelialization [63]. Thus, depending on the type of wound healing or stage of the process, PRP activated using SMLEF bipolar pulses or SMHEF monopolar pulses with higher EGF levels may induce different effects than PRP activated using other methods.

The first study of platelet activation using bipolar electric pulses (bipolar SMLEF pulses in our case) presented here introduces novel and interesting opportunities for appropriately controlling various platelet activation markers. Experimental results shown in the present paper as summarized in Table 1, present capabilities on having select platelet activation markers at levels similar to bovine thrombin (using bipolar SMLEF pulses) or at levels significantly different compared to bovine thrombin (using monopolar SMHEF pulses). Our study should motivate the bioelectrics community to further explore the effects of bipolar SMLEF pulses, considering that higher intensity bipolar pulses have been typically utilized so far in research. Our future research will explore the effects on platelet activation of SMLEF bipolar vs. SMLEF monopolar pulses, with the same voltage, current amplitude and pulse to pulse spacing. The pulse to pulse spacing within a pair of bipolar pulses is about 650 ns for the present work (Fig 1A). The present version of our instrument for platelet activation has been designed for a pulse to pulse spacing of 1 s, for monopolar pulses in conductive coupling mode; bipolar pulses are obtained here by using capacitive coupling, with monopolar pulses via conductive coupling. An improved version of our instrument will hopefully allow for monopolar pulse to pulse spacing similar to bipolar pulses in capacitive coupling. Finally, wound healing studies may quantify the effects of the gentle, more thrombin-like platelet activation via bipolar SMLEF pulses versus the typical monopolar SMHEF pulse treatment [13,15,37].

## Supporting Information

S1 Table Platelet derived microparticles (% of total CD41/Cd42b double positive particles). (DOCX)

S2 Table Percentage of PDMP positive for surface phosphatidylserine as detected by annexin V binding. (DOCX)

S3 Table Percentage of platelets positive for surface phosphatidylserine as detected by annexin V binding. (DOCX)

S4 Table Percentage of all CD41/CD42b double positive particles positive for surface P-selectin. (DOCX)

S5 Table P-selectin mean fluorescence intensity (MFI) per particle. (DOCX)

S6 Table PDGF pg/mL, Lower limit of detection 156.25. (DOCX)

S7 Table VEGF, pg/mL Lower limit of detection, 62.5 pg/mL. (DOCX)

S8 Table Platelet Factor 4, μg/mL. (DOCX)

S9 Table EGF, pg/mL. (DOCX)

S10 Table Normalized cell proliferation. (DOCX)
