# Supplementary material for: Modification of Pulsed Electric Field Conditions Results in Distinct Activation Profiles of Platelet-Rich Plasma
Source: PLoS One. 2016 Aug 24;11(8):e0160933. doi: 10.1371/journal.pone.0160933 (PMC4996457; doi:10.1371/journal.pone.0160933)
Supplement: S1 Table — (DOCX) [file pone.0160933.s001.docx]

**Modification of Pulsed Electric Field Conditions Results in Distinct Activation Profiles of Platelet-rich Plasma**

Andrew L. Frelinger III, Anja J. Gerrits, Allen L. Garner, Andrew S. Torres, Antonio Caiafa, Christine A. Morton, Michelle A. Berny-Lang, Sabrina L. Carmichael, V. Bogdan Neculaes, Alan D. Michelson

**Supporting information:**

**S1 Table.** Platelet derived microparticles (% of total CD41/Cd42b double positive particles)

|  | SMHEF monopolar | SMLEF bipolar | Bov. Thrombin | Vehicle Control |
| --- | --- | --- | --- | --- |
| Donor 1 | 78.5 | 33.7 | 74.4 | 0.6 |
| Donor2 | 54.7 | 53.1 | 41.8 | 0.5 |
| Donor3 | 59.4 | 61.1 | 59.6 | 0.8 |
| Donor4 | 61.8 | 18.6 | 77.2 | 0.8 |
| Donor5 | 64.6 | 15.4 | 39.2 | 1.4 |
