# Supplementary material for: Modification of Pulsed Electric Field Conditions Results in Distinct Activation Profiles of Platelet-Rich Plasma
Source: PLoS One. 2016 Aug 24;11(8):e0160933. doi: 10.1371/journal.pone.0160933 (PMC4996457; doi:10.1371/journal.pone.0160933)
Supplement: S2 Table — (DOCX) [file pone.0160933.s002.docx]

**Modification of Pulsed Electric Field Conditions Results in Distinct Activation Profiles of Platelet-rich Plasma**

Andrew L. Frelinger III, Anja J. Gerrits, Allen L. Garner, Andrew S. Torres, Antonio Caiafa, Christine A. Morton, Michelle A. Berny-Lang, Sabrina L. Carmichael, V. Bogdan Neculaes, Alan D. Michelson

**Supporting information:**

**S2 Table.** Percentage of PDMP positive for surface phosphatidylserine as detected by annexin V binding

|  | SMHEF monopolar | SMLEF bipolar | Bov. Thrombin | Vehicle Control |
| --- | --- | --- | --- | --- |
| Donor 1 | 99.8 | 85.3 | 92.3 | 9.1 |
| Donor2 | 99.7 | 87.8 | 98.4 | 23.1 |
| Donor3 | 99.7 | 72.6 | 79.9 | 31.6 |
| Donor4 | 99.5 | 83.1 | 96.1 | 31.3 |
| Donor5 | 98.3 | 87.8 | 91.8 | 38.2 |
