# Supplementary material for: Modification of Pulsed Electric Field Conditions Results in Distinct Activation Profiles of Platelet-Rich Plasma
Source: PLoS One. 2016 Aug 24;11(8):e0160933. doi: 10.1371/journal.pone.0160933 (PMC4996457; doi:10.1371/journal.pone.0160933)
Supplement: S3 Table — (DOCX) [file pone.0160933.s003.docx]

**Modification of Pulsed Electric Field Conditions Results in Distinct Activation Profiles of Platelet-rich Plasma**

Andrew L. Frelinger III, Anja J. Gerrits, Allen L. Garner, Andrew S. Torres, Antonio Caiafa, Christine A. Morton, Michelle A. Berny-Lang, Sabrina L. Carmichael, V. Bogdan Neculaes, Alan D. Michelson

**Supporting information:**

**S3 Table.** Percentage of platelets positive for surface phosphatidylserine as detected by annexin V binding

|  | SMHEF monopolar | SMLEF bipolar | Bov. Thrombin | Vehicle Control |
| --- | --- | --- | --- | --- |
| Donor 1 | 97.8 | 29.1 | 70.0 | 0.4 |
| Donor2 | 98.2 | 53.8 | 82.2 | 4.4 |
| Donor3 | 99.5 | 50.5 | 71.7 | 19.4 |
| Donor4 | 99.0 | 31.6 | 94.8 | 4.2 |
| Donor5 | 93.9 | 54.8 | 61.5 | 8.9 |
