# Supplementary material for: Modification of Pulsed Electric Field Conditions Results in Distinct Activation Profiles of Platelet-Rich Plasma
Source: PLoS One. 2016 Aug 24;11(8):e0160933. doi: 10.1371/journal.pone.0160933 (PMC4996457; doi:10.1371/journal.pone.0160933)
Supplement: S4 Table — (DOCX) [file pone.0160933.s004.docx]

**Modification of Pulsed Electric Field Conditions Results in Distinct Activation Profiles of Platelet-rich Plasma**

Andrew L. Frelinger III, Anja J. Gerrits, Allen L. Garner, Andrew S. Torres, Antonio Caiafa, Christine A. Morton, Michelle A. Berny-Lang, Sabrina L. Carmichael, V. Bogdan Neculaes, Alan D. Michelson

**Supporting information:**

**S4 Table.** Percentage of all CD41/CD42b double positive particles positive for surface P-selectin

|  | SMHEF monopolar | SMLEF bipolar | Bov. Thrombin | Vehicle Control |
| --- | --- | --- | --- | --- |
| Donor 1 | 55.97 | 78.10 | 83.34 | 20.16 |
| Donor2 | 55.52 | 67.51 | 63.95 | 27.53 |
| Donor3 | 38.71 | 71.77 | 70.57 | 34.10 |
| Donor4 | 42.72 | 84.58 | 75.79 | * |
| Donor5 | 42.30 | 76.95 | 67.20 | 18.46 |

*technical error, no data available.
