# Supplementary material for: Modification of Pulsed Electric Field Conditions Results in Distinct Activation Profiles of Platelet-Rich Plasma
Source: PLoS One. 2016 Aug 24;11(8):e0160933. doi: 10.1371/journal.pone.0160933 (PMC4996457; doi:10.1371/journal.pone.0160933)
Supplement: S5 Table — (DOCX) [file pone.0160933.s005.docx]

**Modification of Pulsed Electric Field Conditions Results in Distinct Activation Profiles of Platelet-rich Plasma**

Andrew L. Frelinger III, Anja J. Gerrits, Allen L. Garner, Andrew S. Torres, Antonio Caiafa, Christine A. Morton, Michelle A. Berny-Lang, Sabrina L. Carmichael, V. Bogdan Neculaes, Alan D. Michelson

**Supporting information:**

**S5 Table.** P-selectin mean fluorescence intensity (MFI) per particle

|  | SMHEF monopolar | SMLEF bipolar | Bov. Thrombin | Vehicle Control |
| --- | --- | --- | --- | --- |
| Donor 1 | 9.91 | 30.23 | 25.35 | 4.20 |
| Donor2 | 12.05 | 21.72 | 22.04 | 6.06 |
| Donor3 | 7.60 | 24.35 | 20.61 | 6.95 |
| Donor4 | 8.35 | * | 24.38 | * |
| Donor5 | 6.46 | 76.67 | 19.49 | 3.64 |

*technical error, no data available.
