# Supplementary material for: Modification of Pulsed Electric Field Conditions Results in Distinct Activation Profiles of Platelet-Rich Plasma
Source: PLoS One. 2016 Aug 24;11(8):e0160933. doi: 10.1371/journal.pone.0160933 (PMC4996457; doi:10.1371/journal.pone.0160933)
Supplement: S6 Table — (DOCX) [file pone.0160933.s006.docx]

**Modification of Pulsed Electric Field Conditions Results in Distinct Activation Profiles of Platelet-rich Plasma**

Andrew L. Frelinger III, Anja J. Gerrits, Allen L. Garner, Andrew S. Torres, Antonio Caiafa, Christine A. Morton, Michelle A. Berny-Lang, Sabrina L. Carmichael, V. Bogdan Neculaes, Alan D. Michelson

**Supporting information:**

**S6 Table.** PDGF pg/mL, Lower limit of detection 156.25

|  | SMHEF monopolar | SMLEF bipolar | Bov. Thrombin | Vehicle |
| --- | --- | --- | --- | --- |
| Donor 1 | 9703.3 | 13132.7 | 10456.8 | 156.25 |
| Donor 2 | 9753.5 | 9489.9 | 12015.9 | 235.80 |
| Donor 3 | 7693.8 | 13282.6 | 10134.7 | 361.00 |
| Donor 4 | 15643.3 | 26552.4 | 25586.4 | 567.50 |
| Donor 5 | 12751.0 | 12990.6 | 12535.7 | 261.80 |
