# Supplementary material for: Modification of Pulsed Electric Field Conditions Results in Distinct Activation Profiles of Platelet-Rich Plasma
Source: PLoS One. 2016 Aug 24;11(8):e0160933. doi: 10.1371/journal.pone.0160933 (PMC4996457; doi:10.1371/journal.pone.0160933)
Supplement: S7 Table — (DOCX) [file pone.0160933.s007.docx]

**Modification of Pulsed Electric Field Conditions Results in Distinct Activation Profiles of Platelet-rich Plasma**

Andrew L. Frelinger III, Anja J. Gerrits, Allen L. Garner, Andrew S. Torres, Antonio Caiafa, Christine A. Morton, Michelle A. Berny-Lang, Sabrina L. Carmichael, V. Bogdan Neculaes, Alan D. Michelson

**Supporting information:**

**S7 Table.** VEGF, pg/mL Lower limit of detection, 62.5 pg/mL

|  | SMHEF monopolar | SMLEF bipolar | Bov. Thrombin | Vehicle |
| --- | --- | --- | --- | --- |
| Donor 1 | 1291.3 | 1427.8 | 1283.5 | 62.5 |
| Donor 2 | 630.4 | 688.5 | 564.0 | 62.5 |
| Donor 3 | 931.4 | 918.5 | 704.5 | 62.5 |
| Donor 4 | 603.2 | 691.1 | 548.0 | 62.5 |
| Donor 5 | 412.0 | 191.8 | 64.2 | 62.5 |
