# Supplementary material for: Modification of Pulsed Electric Field Conditions Results in Distinct Activation Profiles of Platelet-Rich Plasma
Source: PLoS One. 2016 Aug 24;11(8):e0160933. doi: 10.1371/journal.pone.0160933 (PMC4996457; doi:10.1371/journal.pone.0160933)
Supplement: S8 Table — (DOCX) [file pone.0160933.s008.docx]

**Modification of Pulsed Electric Field Conditions Results in Distinct Activation Profiles of Platelet-rich Plasma**

Andrew L. Frelinger III, Anja J. Gerrits, Allen L. Garner, Andrew S. Torres, Antonio Caiafa, Christine A. Morton, Michelle A. Berny-Lang, Sabrina L. Carmichael, V. Bogdan Neculaes, Alan D. Michelson

**Supporting information:**

**S8 Table.** Platelet Factor 4, µg/mL

|  | SMHEF monopolar | SMLEF bipolar | Bov. Thrombin | Vehicle |
| --- | --- | --- | --- | --- |
| Donor 1 | 24.65 | 29.20 | 21.06 | 0.43 |
| Donor 2 | 20.56 | 14.86 | 18.14 | 0.46 |
| Donor 3 | 8.88 | 15.49 | 16.41 | 0.25 |
| Donor 4 | 9.87 |  | 30.00 | 0.29 |
| Donor 5 | 10.22 | 13.06 | 15.04 | 0.28 |
