# Supplementary material for: Modification of Pulsed Electric Field Conditions Results in Distinct Activation Profiles of Platelet-Rich Plasma
Source: PLoS One. 2016 Aug 24;11(8):e0160933. doi: 10.1371/journal.pone.0160933 (PMC4996457; doi:10.1371/journal.pone.0160933)
Supplement: S9 Table — (DOCX) [file pone.0160933.s009.docx]

**Modification of Pulsed Electric Field Conditions Results in Distinct Activation Profiles of Platelet-rich Plasma**

Andrew L. Frelinger III, Anja J. Gerrits, Allen L. Garner, Andrew S. Torres, Antonio Caiafa, Christine A. Morton, Michelle A. Berny-Lang, Sabrina L. Carmichael, V. Bogdan Neculaes, Alan D. Michelson

**Supporting information:**

**S9 Table.** EGF, pg/mL

|  | SMHEF monopolar | SMLEF bipolar | Bov. Thrombin | Vehicle |
| --- | --- | --- | --- | --- |
| Donor 1 | 3930.19 | 2378.68 | 2004.91 | 19.62 |
| Donor 2 | 2624.34 | 2476.60 | 1106.04 | 33.21 |
| Donor 3 | 2900.94 | 3228.30 | 1649.62 | 10.94 |
| Donor 4 | 2284.91 | 1512.45 | 1218.49 | 7.17 |
| Donor 5 | 2750.19 | 2217.36 | 1149.06 | 14.72 |
