# Supplementary material for: Modification of Pulsed Electric Field Conditions Results in Distinct Activation Profiles of Platelet-Rich Plasma
Source: PLoS One. 2016 Aug 24;11(8):e0160933. doi: 10.1371/journal.pone.0160933 (PMC4996457; doi:10.1371/journal.pone.0160933)
Supplement: S10 Table — (DOCX) [file pone.0160933.s010.docx]

**Modification of Pulsed Electric Field Conditions Results in Distinct Activation Profiles of Platelet-rich Plasma**

Andrew L. Frelinger III, Anja J. Gerrits, Allen L. Garner, Andrew S. Torres, Antonio Caiafa, Christine A. Morton, Michelle A. Berny-Lang, Sabrina L. Carmichael, V. Bogdan Neculaes, Alan D. Michelson

**Supporting information:**

**S10 Table.** Normalized cell proliferation

|  | SMHEF monopolar | SMLEF bipolar | Bov. Thrombin | Vehicle Control |
| --- | --- | --- | --- | --- |
| Donor 1 | 1.095 | 1.266 | 1.138 | 0.958 |
| Donor2 | 1.190 | 1.361 | 1.363 | 1.039 |
| Donor3 | 1.074 | 1.155 | 1.059 | 0.906 |
| Donor4 | 1.229 | 1.069 | 1.099 | 1.121 |
| Donor5 | 1.347 | 1.138 | 1.064 | 0.973 |
